# How path integration abilities of blind people change in different exploration conditions

**DOI:** 10.3389/fnins.2024.1375225

**Published:** 2024-05-17

**Authors:** Shehzaib Shafique, Walter Setti, Claudio Campus, Silvia Zanchi, Alessio Del Bue, Monica Gori

**Affiliations:** ^1^Unit of Visually Impaired People (U-VIP), Italian Institute of Technology, Genova, Italy; ^2^Pattern Analysis and Computer Vision (PAVIS), Italian Institute of Technology, Genova, Italy

**Keywords:** blind navigation, path integration, shape completion, triangle completion task, spatial navigation, environment encoding, guided condition, feedback preference

## Abstract

For animals to locate resources and stay safe, navigation is an essential cognitive skill. Blind people use different navigational strategies to encode the environment. Path integration significantly influences spatial navigation, which is the ongoing update of position and orientation during self-motion. This study examines two separate things: (i) how guided and non-guided strategies affect blind individuals in encoding and mentally representing a trajectory and (ii) the sensory preferences for potential navigational aids through questionnaire-based research. This study first highlights the significant role that the absence of vision plays in understanding body centered and proprioceptive cues. Furthermore, it also underscores the urgent need to develop navigation-assistive technologies customized to meet the specific needs of users.

## 1 Introduction

Spatial representation is a cognitive skill involved in the storage and processing of spatial information, enabling individuals to comprehend their environment and the spatial relationships among its elements and to carry out day-to-day activities (Kosslyn and Osherson, 1995; Dennis and Tapsfield, 2013; Grieves and Jeffery, 2017). For individuals with intact visual sensory abilities, both visual and non-visual inputs such as vestibular and proprioceptive cues play a significant role in navigation (Bates and Wolbers, 2014) even though vision is pivotal in this context (Shelton and Yamamoto, 2009; Ekstrom, 2015). Consequently, those with visual impairments encounter difficulties when navigating unfamiliar environments such as bus stops, malls, and offices (Khusro et al., 2022). Studies indicate that blind individuals may have reduced skills related to inferential navigation (Seemungal et al., 2007) and lower sensitivity to perspective shifts (Rieser et al., 1986). In contrast to sighted individuals, they often exhibit slower walking, adopt a cautious posture, take shorter strides, and have longer stance times (Massiceti et al., 2018). In fact, without vision, there are fewer options for processing spatial information, thus necessitating a more extensive use of executive cognitive functions (Ruggiero and Iachini, 2010). Vision uniquely enables the simultaneous perception and manipulation of multiple pieces of information, whereas alternative sensory modalities like touch and hearing predominantly relied on by blind individuals typically permit only serial processing of spatial information (Setti et al., 2018; Amadeo et al., 2019).

The absence of visual input in blind individuals necessitates the use of alternative sensory modalities and cognitive strategies for encoding spatial layouts and navigating through environments. Researches have shown that blind individuals often compensate for the lack of visual information by enhancing their use of auditory, tactile, and olfactory cues, as well as developing sophisticated mechanisms for spatial cognition and memory (Likova and Cacciamani, 2018; Santoro et al., 2020). When vision is absent or impaired, auditory cues play a pivotal role in providing essential information for orientation and movement, such as depth and distance from objects (Wiener and Lawson, 1997; Koutsoklenis and Papadopoulos, 2011). For instance, echoes are one type of auditory signal that can assist in maintaining direction within a certain region and provide crucial information about landmarks and objects for visually impaired people (Picinali et al., 2014). Blind individuals frequently switch between different encoding techniques, underscoring the adaptability of their navigation strategies. Comprehending the intricate interactions among these adaptive strategies, sensory input, and cognitive processes is crucial for understanding the challenges that blind individuals encounter when navigating their surroundings.

To study spatial navigation abilities in blind or sighted people, path integration tasks are usually carried out (Bredin et al., 2005; Xie et al., 2017; Anson et al., 2019; Dorado et al., 2019; Harootonian et al., 2020). They test participants' skills to move and learn spatial layouts. Using the path integration cognitive process, an organism gathers and organizes data about its trajectory in relation to a starting position. This complex navigational skill is based on the continuous updating and determination of the organism's current position in space through the use of idiothetic signals (Mittelstaedt, 1999). The existing literature on path integration has primarily focused on studying blind individuals or blindfolded sighted individuals. The comparison between blindfolded sighted and blind individuals allow us to understand the differences in the mechanisms and strategies used according to the transient vs permanent absence of visual cues. It is known that sighted individuals have a better experience of path integration than visually impaired people (Brambring, 1976). However, differences in the performances have been observed, between sighted and blind individuals, based on the strategies opted to encode the environment. For example, several studies have found little or no performance differences between sighted and blind persons (Tinti et al., 2006; Rogge et al., 2021). Moreover, there were studies where sighted individuals showed more errors than blind persons (Blanco and Travieso, 2003; Iachini and Ruggiero, 2010).

Regarding this specific task, performance evaluations usually entail evaluating participants according to how much the path's starting point differs from where they arrive (Xie et al., 2017). The degree to which participants' final positions align with the planned trajectory's starting point serves as an approximation for the participants' efficiency. The triangle completion task is the most used in this context. In its traditional procedure, the experimenter leads the subjects on two sides of a triangular path before asking them to walk the third side of the triangle and stop when reaching the initial point to close the loop (Garcia et al., 2015).

Besides the triangular completion task, some authors used more complex shapes to study blind people's navigational abilities (Iachini and Ruggiero, 2010; Koutakis et al., 2013; Gori et al., 2017). Gori et al. (2017) used 30 different shapes, including circles, squares, and triangles, to evaluate the abilities of blind individuals to integrate motor and perceptual information. They also tested the influence of prior visual experience in these processes by comparing the performances of early- and late-onset blind people. The results showed a lack of performance of early blinds in comparison to the sighted. Moreover, Koutakis et al. (2013) included blindfolded walking for path integration to highlight the roles of sensory inputs using circular paths and figure-eight paths. Koutakis concluded that the greater the complexity, the greater the error in path integration and more reliance on external feedback. These studies provide insight into the spatial difficulties blind people encounter when traversing intricate situations. These investigations clarified the difficulties blind individuals encounter when integrating motor and perceptual information, cognitively picturing large-scale settings, and completing path integration by utilizing a variety of forms and paths. Their results highlight how difficult it is to navigate without visual cues based on the complexity and strategy used for path integration.

Blind individuals encounter the daunting task of navigating through complex built environments, which can be confusing, disorienting, and overwhelming (Imrie and Hall, 2003). Studies focusing on visual impairment and blindness aim to delve into the cognitive mechanisms involved in non-visual navigation while also striving to devise assistive technologies to aid in avoiding obstacles and selecting optimal routes. Based on the previous findings of path integration, many navigational devices have been developed [for a thorough discussion of these systems, please see the review of Kuriakose et al. (2022)]. The primary goal of every navigational aid is to provide adequate information so that a person can reach his destination. According to Ran et al. (2004), numerous navigation systems for blind and visually impaired people have been proposed, but only a small number of these systems can offer dynamic interactions and flexibility to changes, and none of them operate flawlessly indoors and outdoors. Current navigational aids are not widely accepted by blind individuals (Loomis et al., 2018) due to several reasons: some navigational aids are built to help the exploration only for specific environments (Schwarze et al., 2016), the equipment is often expensive (Chaccour and Badr, 2015), some of the tools report poor accuracy to detect objects (Bai et al., 2017) and are difficult to use (Marder-Eppstein, 2016). Among these factors, the main reason for abandoning assistive technologies is neglecting users' opinions in the development process (Kuriakose et al., 2022). These drawbacks highlight the demand for a more adaptable navigation system designed to meet the particular requirements of the blind and visually impaired community (Chaccour and Badr, 2015; Marder-Eppstein, 2016; Schwarze et al., 2016; Bai et al., 2017).

In this work, two separate objectives were achieved. Firstly, we looked into how different navigational strategies used by blind people help to encode the environment and affect spatial navigation. In particular, we studied how blind people navigate when they follow the guidance of another person (guided condition) as opposed to when they move alone, relying just on auditory cues for direction (non-guided condition). Based on previous studies (Beni and Cornoldi, 1988; Cattaneo et al., 2008), we hypothesized that blind people perform better in the guided condition of spatial navigation than in non-guided condition. Moreover, to assess whether differences in navigation performance of blind people are due to inherent abilities or simply the result of using different strategies to complete the tasks, we considered blindfolded sighted people in our study and tested them. Secondly, to understand the problems faced by blind people related to current navigational aids and their needs for future navigational aids, a thorough questionnaire was administered.

## 2 Methodology

### 2.1 Participants

The present research study involved the participation of twenty visually impaired individuals (mean age: 42.32 ± 12.25 years, 10 females) and nine blindfolded sighted individuals (mean age: 40.34 ± 11.20 years, 6 females). All twenty participants completed the administered questionnaire, but only 13 out of 20 participated in the experimental study. Among the total twenty blind participants, only 13 participants took part in the experiment (mean age: 46.79 ± 12.30, 7 females). All blind participants had no other sensory or motor impairment.

The research study obtained informed written consent from every participant, and all experiments were carried out following the Helsinki Declaration. The experiments received approval by the local health service ethics committee (Comitato Etico, ASL 3, Genoa, Italy). As the duration of the session varied based on the participant's abilities, the time of the whole session was flexible but generally lasted for around 45 min.

The participants were called from the database of the Italian Institute of Technology. All participants were of Italian nationality. The clinical history of the blind participants who participated in the experimental study can be found in Table 1. From Table 1, the first thirteen are those participants who also took part in the experimental study.

**Table 1:** Clinical details of blind subjects who participated in the study.

**ID**	**Gender**	**Type of blind**	**Age (years)**	**Pathology**	**Blindness onset**
S1	Female	Late blind	46	Accident, loss of retina	18 years
S2	Female	Early blind	33.5	Retinopathy of prematurity	Before birth
S3	Female	Early blind	34.3	ICD9–362.7 retinitis pigmentosa	Before birth
S4	Male	Late blind	47.2	ICD9–362.7 retinitis pigmentosa	34 years
S5	Male	Late blind	63	Uveitis	11 years
S6	Female	Late blind	57.8	Rystagmus and retinitis pigmentosa	30–40 years
S7	Female	Early blind	28.9	Congenital glaucoma and retinal detachment	Before birth
S8	Male	Early blind	54.1	Benign tumor in optic nerve	Birth
S9	Male	Early blind	57.3	Retinopathy of prematurity	Birth
S10	Male	Early blind	30.8	Leber congenital amaurosis	Birth
S11	Male	Early blind	50.6	Congenital atrophy of Optic nerve	Birth
S12	Female	Early blind	40.5	Congenital cataract/attic atrophy	Birth
S13	Female	Early blind	64.3	Microstalmica	Birth
S14	Male	Late blind	30.1	Stargardt disease	12 years
S15	Male	Late blind	63.4	Degenerative myopic maculopathy	8 years
S16	Male	Early blind	54.4	Microstalmica	Birth
S17	Female	Late blind	65.3	Leber syndrome	27 years
S18	Female	Early blind	47.3	Congenital Cataract/Attic Atrophy	Birth
S19	Male	Early blind	36.5	Retinopathy of Prematurity	Birth
S20	Female	Late Blind	36.5	Bilateral Glaucoma	20 years

### 2.2 Experimental procedure

The procedure was split into two sections: the navigation task and the questionnaire administration. The details of these sections are described below.

#### 2.2.1 Shape completion task

The data was collected in a quiet room (length = 5.36 m and width = 6.37 m). Both blindfolded sighted and blind participants participated in the experimental study. Before performing the experiment, we described to our participants the features of the room environment where we conducted the experiment. None of the participants was allowed to use a cane. Participants were instructed to undertake a path integration task involving two trajectories: a straight and a triangular path. The length of the straight path was 3.5102 m. The length of the triangular path leg of Point A to Point B, Point B to Point C, and Point C to Point A were 3.8744 m, 3.7771 m, and 3.1268 m, respectively. The approximate angles at vertex A, B, and C were 53.7, 61.2, and 65.1 degrees, respectively. Each trajectory had two conditions that were counterbalanced across the participants: guided (the experimenter helps in the exploration of the environment) and non-guided (autonomous exploration of the environment) spatial navigation techniques. The primary objective of this task was to assess the impact of a guided and non-guided exploration strategy on the ability of visually impaired individuals to learn a spatial layout.

**Figure 1:**
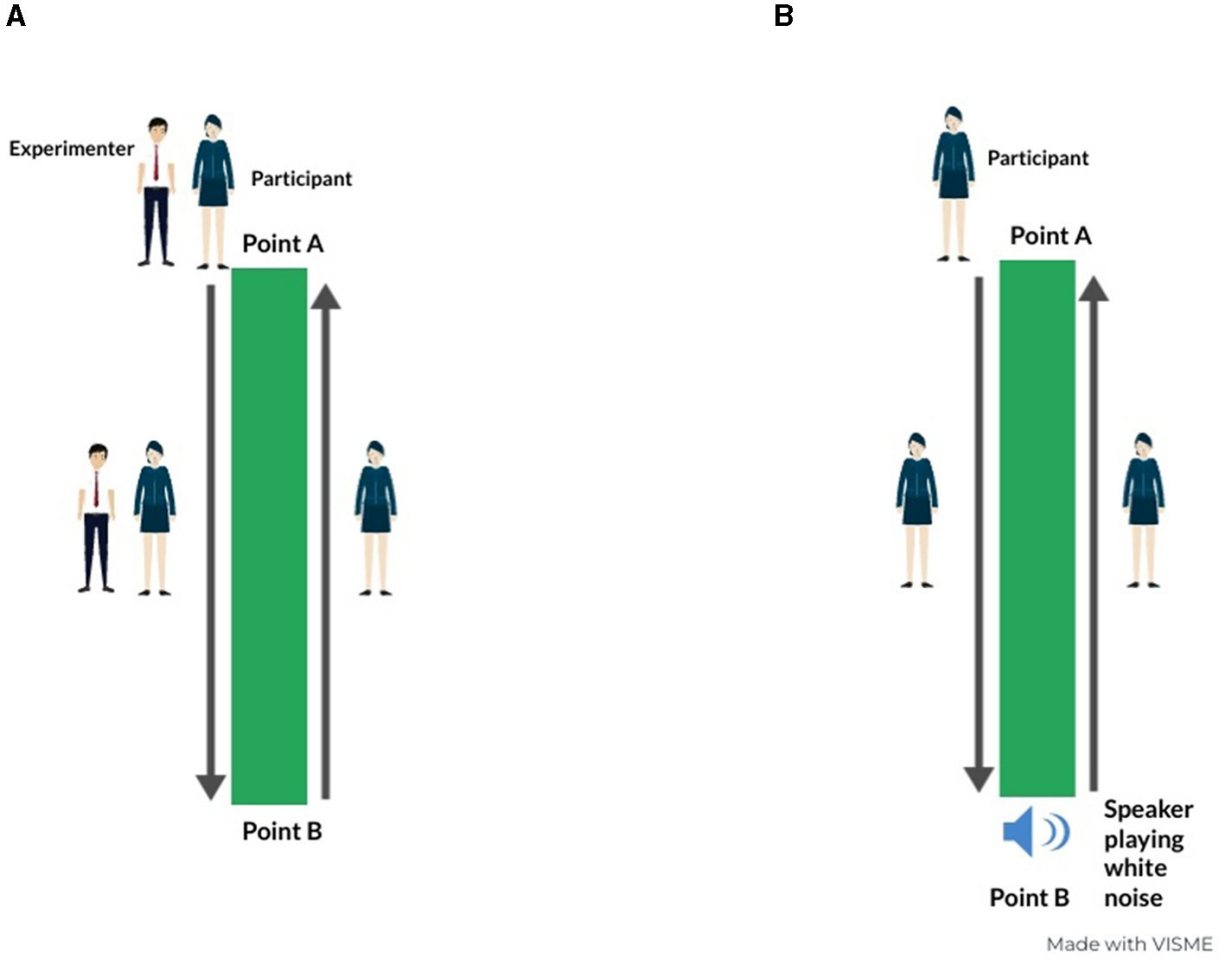
Visual representation of guided **(A)** and non-guided **(B)** conditions of the straight path. This infographic is made using viseme.co.

In the guided condition of the straight path, the experimenter gently guided the participant from point A to B, as depicted on the left panel of Figure 1. Subsequently, the participant was tasked with returning to the initial position unaided. Conversely, in the non-guided condition of the straight path, a speaker playing white noise was placed on point B until the participant reached the point, serving as a spatial reference point, i.e., a landmark. Landmarks play a crucial role in providing navigational cues and facilitating the development of a spatial representation of the surrounding environment (Samany et al., 2009). In this case, the participants, starting from point A, reached the point B by following the sound played by the speaker. Once they reached point B, the speaker stopped playing white noise and they retraced their steps to point A, mirroring the procedure in the passive phase (see the right panel of Figure 1).

**Figure 2:**
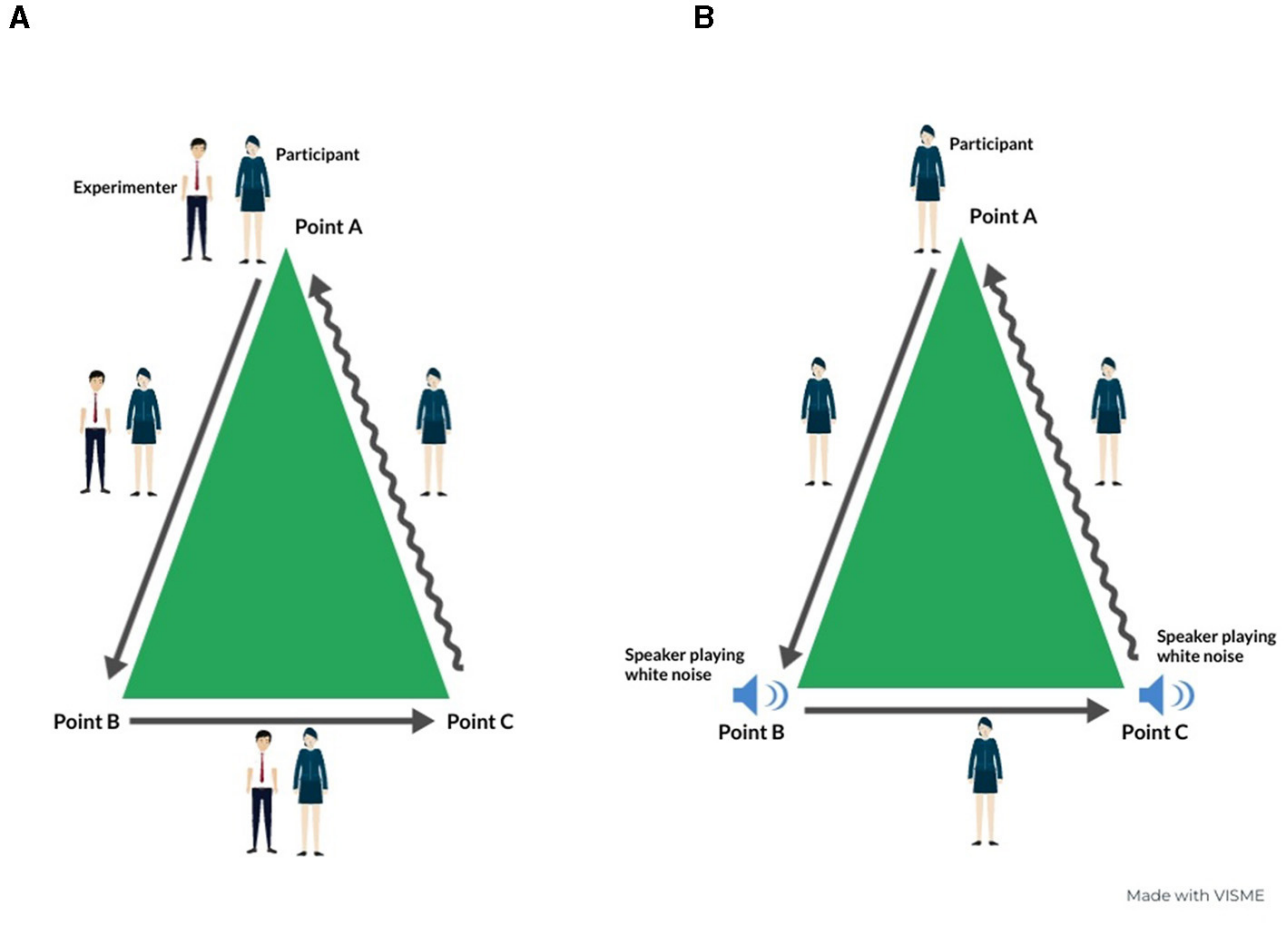
Visual representation of guided **(A)** and non-guided **(B)** condition of the triangular path. This infographic is made using viseme.co.

In the guided exploration condition of the triangular path, the experimenter initiated the journey from point A and guided the participant along the two sides of the triangle, passing through points B and C (Figure 2A). At each vertex, they paused briefly to emphasize the participant's location on the vertex. Following this, the participant was instructed to return to the initial position autonomously, with the task of completing the triangular loop inferring the third side. These specific steps and instructions are depicted in the left panel of Figure 2. Conversely, in the non-guided condition of the triangular path, two speakers emitting white noise were positioned at points B and C (Figure 2B). These speakers were activated one at a time and continuously played sound until the participants reached their respective locations. In detail, when the subject reached point B, the white noise stopped. Then, the white noise from the speaker at point C started, and the participant moved toward that point. Upon reaching point C, the white noise was stopped. The participant was once again directed to return to the initial position autonomously. The non-guided condition of the triangular condition is shown in Figure 2B.

In both conditions, a tape strip was placed in correspondence with the participants' right heel to mark the point where they stopped. From this point, the absolute distance from the actual initial point was calculated in centimeters to determine the participants' error (i.e., how far they stopped from the initial point). Each participant performed three trials for each condition for both phases; thus, twelve trials were conducted in total.

#### 2.2.2 Questionnaire

Each visually impaired participant was given a five-query questionnaire in Italian to gather information on the issues faced while walking, their experience with current technologies for navigation, and their interest in future mobility devices. The ultimate goal of this survey was to understand the real needs and issues of visually impaired people and to use this information to design a novel user-centered technology to help them move around in the surrounding environment. The questionnaire consisted of the following questions:

1. *Preferisci andare in giro da solo o accompagnato?*
2. *Ti trovi a tuo agio con la Braille (i punti in rilievo che raffigurano le lettere dell'alfabeto vengono utilizzati in un metodo di lettura e scrittura tattile per i non vedenti)?*
3. *Normalmente, oltre al bastone, usi qualche altra applicazione per la navigazione?*
4. *Quali limiti trovi agli odierni sistemi di navigazione spaziale disponibili per i non vedenti?*
5. *In futuro svilupperemo un nuovo dispositivo multisensoriale per la navigazione spaziale. Quale feedback sensoriale preferiresti che questo strumento utilizzasse per aiutarti a muoverti, trovare ed evitare ostacoli sul tuo percorso?*

English Translation:

1. Do you prefer to walk around alone or accompanied?
2. Are you comfortable with Braille (raised dots that depict the letters of the alphabet are used in a touch reading and writing method for blind people)?
3. Do you usually use any other navigation application besides the cane?
4. What limits do you find to today's space navigation systems available to blind people?
5. In the future, we will develop a new multisensory device for spatial navigation. Which sensory feedback would you prefer this tool to use in helping you move, find, and avoid obstacles in your path?

### 2.3 Data analysis

All data were analyzed using RStudio 2023.03.0 statistical software. Data obtained from Section 1, which involves the path integration task, we computed the average spatial errors in reaching the final point across the three trials for each condition. Subsequently, we conducted Wilcoxon *t*-tests using the wilcox.test(), from package “stats” (R Core Team, 2013), to compare conditions. In relation to Section 2 (answers to the questionnaire), data were analyzed using a chi-squared test implemented by Chisq.test() function from package “stats” (R Core Team, 2013).

## 3 Results

### 3.1 Results of section 1 (experimental methodology section)

We used a two-condition design, consisting of a “Guided condition” and a “Non-guided condition,” to compare the spatial navigation abilities of blind and sighted individuals. We calculated the absolute error, that is, the Euclidean distance between the actual starting and the final points reached by the participants.

**Figure 3:**
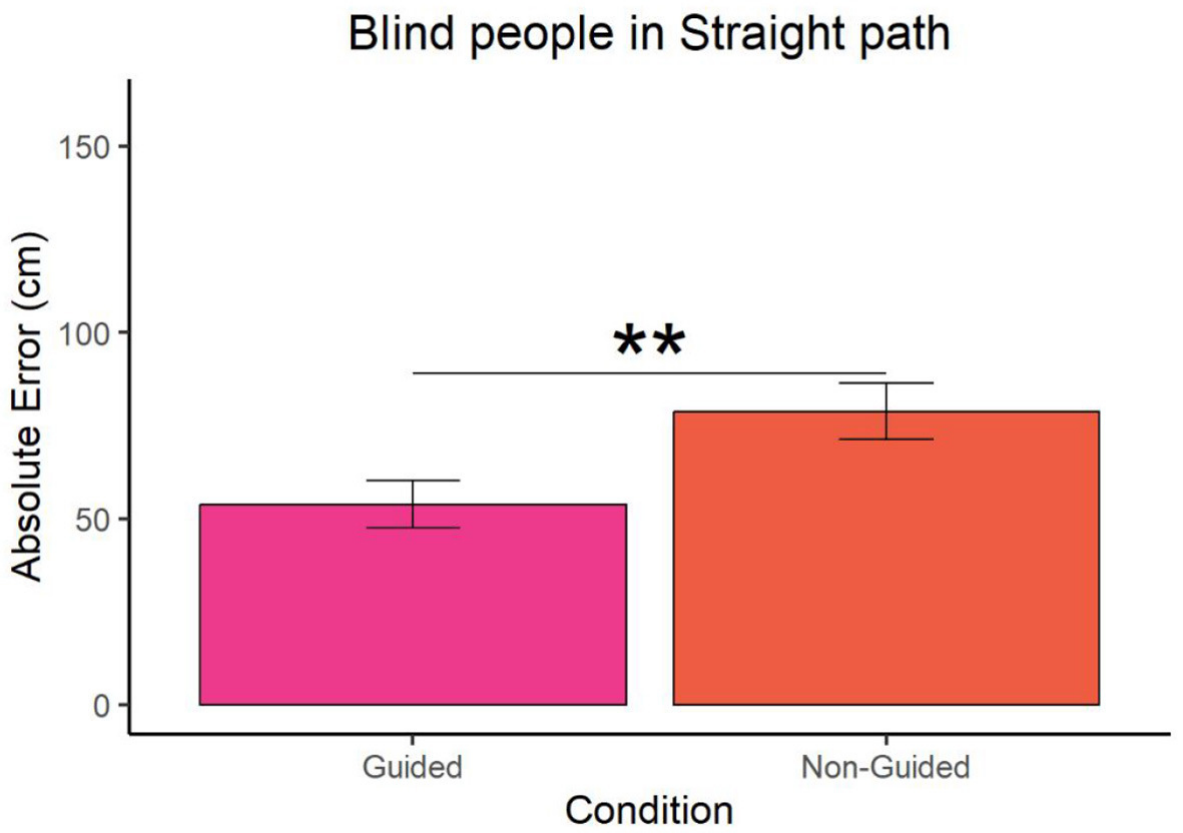
Absolute error of blind participants at point A for the straight path. "^**^" represents a p-value less than 0.01.

**Figure 4:**
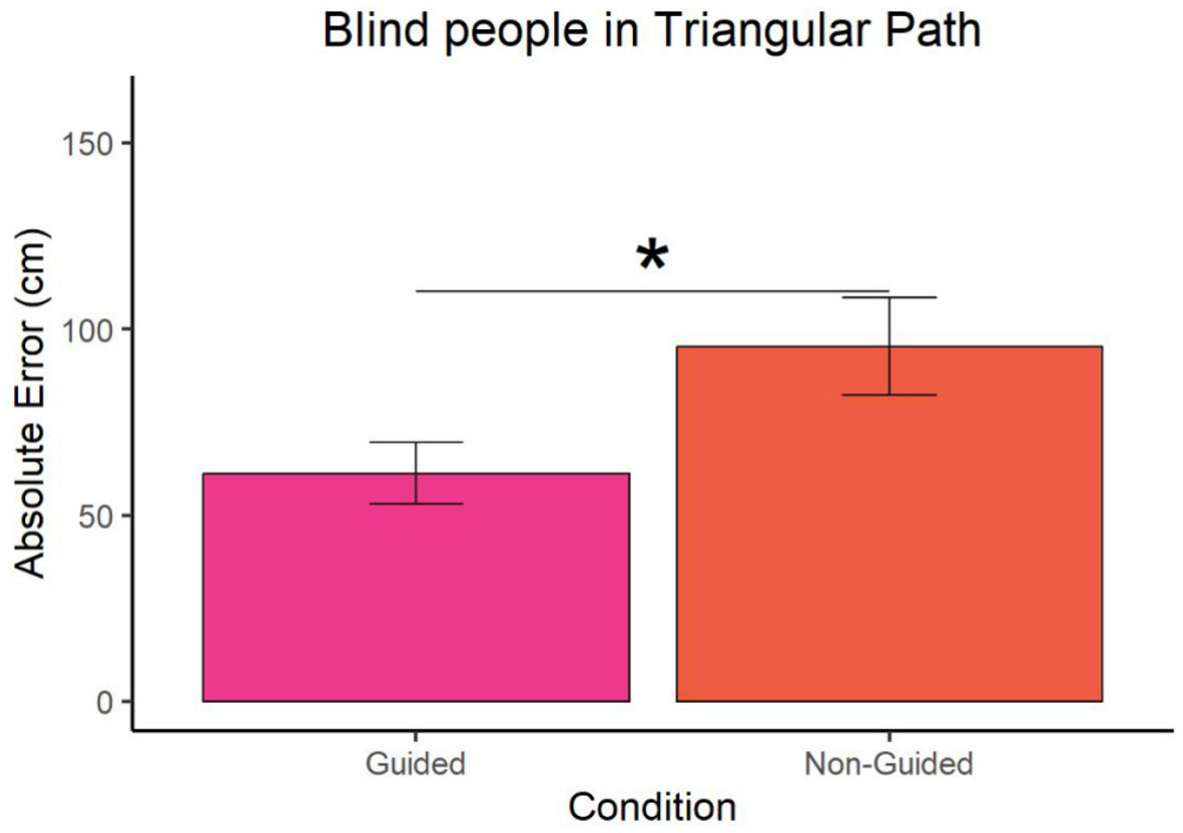
Absolute error of blind participants at point A for the triangular path. "^*^" represents a p-value less than 0.05.

In the case of blind participants, Wilcoxon *t*-test revealed that the error was lower in the guided compared to the non-guided condition for both the straight (W = 41, p-value = 0.007 and ∣*r*∣ = 0.16) and triangular paths (W = 53.5, *p*-value = 0.043 and ∣*r*∣ = 0.27) as shown in Figures 3, 4 respectively. For guided and non-guided conditions, in a straight path, the mean errors were 53.82857 cm and 78.74286 cm, respectively. Similarly, in the triangular path, the mean errors for guided and non-guided conditions were 61.2619 cm and 95.34048 cm, respectively.

**Figure 5:**
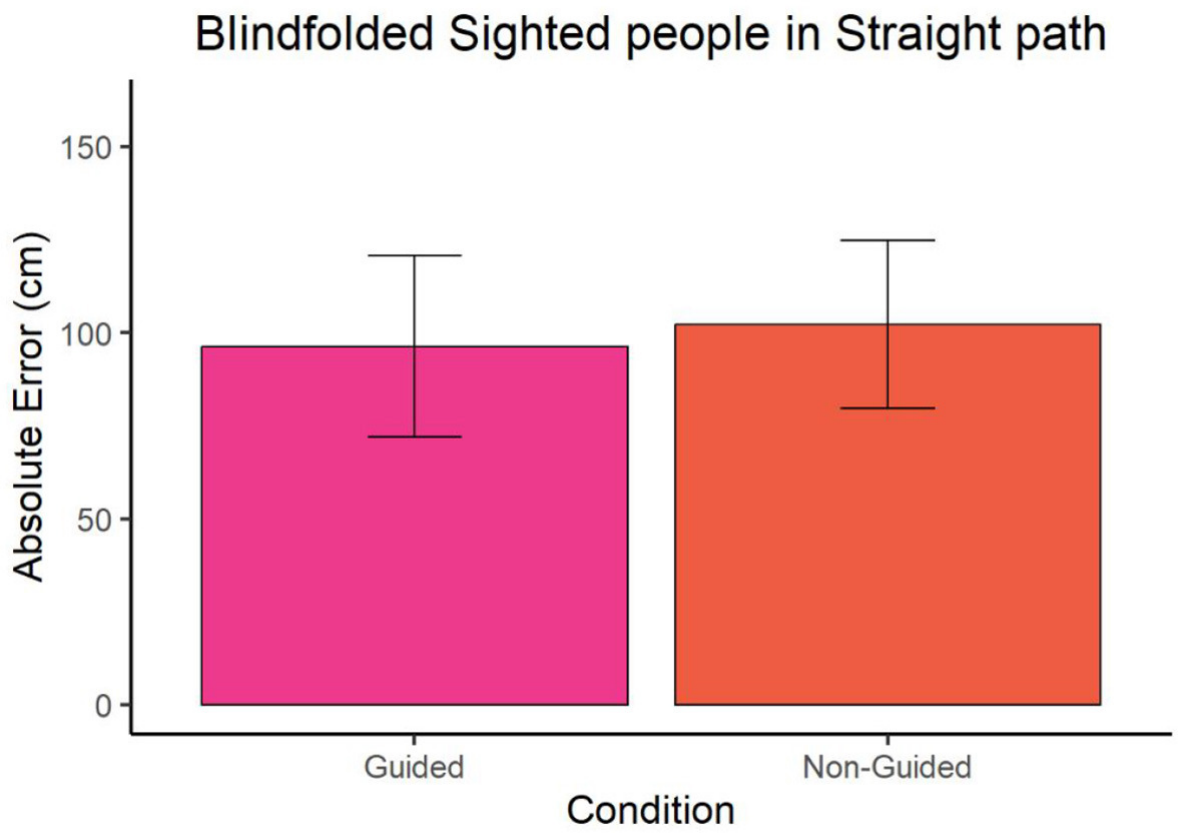
Absolute error of blindfolded sighted participants at point A for the straight path.

**Figure 6:**
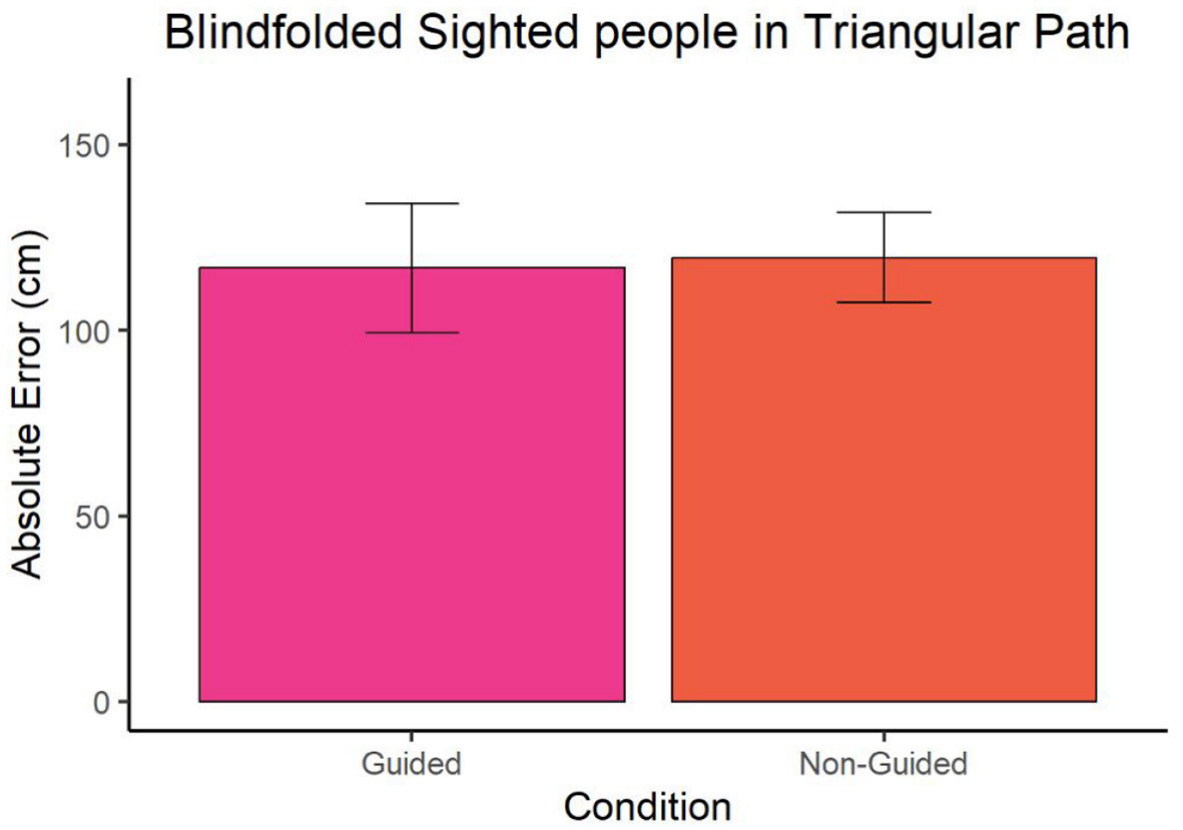
Significance plot of absolute error of blindfolded sighted participants at point A for the triangular path.

In the case of blindfolded sighted participants, we observed no significant difference between the guided and non-guided condition in both straight path (W = 39, *p*-value = 0.9314 and ∣*r*∣ = 0.35) and triangular path (W = 44, *p*-value = 0.7962 and ∣*r*∣ = 0.78) as shown in Figures 5, 6. The mean error in guided and non-guided conditions of the straight path was 96.24 cm and 102.10 cm, respectively. In the triangular path, the mean error in guided and non-guided conditions was 116.71 cm and 119.57 cm, respectively.

### 3.2 Result of section 2 (questionnaire section)

The questionnaire was designed to collect feedback from participants regarding their experiences with current navigation technologies and their requirements for future mobility technology. It comprised five questions encompassing various topics such as motor issues, challenges in daily activities, and the demand for new devices. Upon analyzing the responses, we identified several trends that offer valuable insights into understanding the needs of blind individuals for spatial navigation. When we asked blind people whether they move alone or accompanied and whether they use any navigation applications or just a cane, 61% people said that they move alone without any company, while 39% said they move with the company of any person or dog. The chi-squared results imply no statistically significant difference from fifty percent of the expected frequency (*x*^2^ = 0.8, DF = 1, *p* = 0.37). In addition, no significance was seen between the people using any navigation application and those using only a cane because 50% said they use only a cane and 50% said they use a navigation application (*x*^2^ = 0, DF = 1, *p* = 1). When we asked blind people about their comfort using Braille, 77.77% of subjects voted for using Braille, while 22.22% of subjects did not favor Braille. The chi-squared analysis showed that there is a statistically significant difference between our results and fifty percent expected frequency (*x*^2^ = 7.2, DF= 1, *p* = 0.007). However, our primary and most crucial question was to determine the sensory feedback they would prefer to obtain information about the surrounding environment. From the result, 25% of the people who completed the questionnaire would prefer spatial information to be acquired through the auditory channel, while 75% would prefer it through vibrotactile feedback. The chi-squared analysis showed a significant difference from the expected fifty percent frequency (*x*^2^ = 5, DF = 1, and *p* = 0.025).

Blind participants in our study discussed their problems and experiences with current navigation tools. One participant reported attempting to use a vibrating cane but complaining that the handle was too heavy to use comfortably. Another expressed concern regarding depending solely on navigator-based systems, citing issues with their accuracy, power consumption, and incapacity to guarantee total safety. The oversensitivity of vibrating canes to obstructions, including sidewalks, without offering thorough route direction was an often brought-up concern. Participants also brought up issues, such as signal loss, with GPS applications for space navigation. While beneficial, other devices, such as artificial intelligence-based apps and camera-equipped eyewear, have disadvantages, such as requiring internet access and occupying hands. Some participants felt that specialized sound apps and other sound-based tools provided too much information. Concerns were also expressed regarding the difference between the tactile feedback of vibrating canes and the auditory distraction caused by navigation apps.

## 4 Discussion

Blind people navigate and make sense of the environment by combining different exploration strategies. In this study, divided into two sections, we first investigated which of two exploratory strategies (i.e., guided and non-guided) best helps a blind person to learn spatial trajectories. Secondly, we studied through a 5-queries questionnaire, the problems blind people face while walking with the navigational aids to date available on the market. As far as our experimental results are concerned, our findings suggest that in both straight and triangular paths, only the group of blind participants shows greater performance in the guided condition than in the non-guided one. Different possible reasons can explain these results. First, the designed protocol of the non-guided condition required complex memory processing as the individuals were required to listen to the sound, move toward it, remember their orientation and position in space, and then return to the initial point. Furthermore, in the case of the triangular trajectory, the non-guided condition involved a sequential acquisition of information (Bregman and Pinker, 1978). Participants would start from the initial point and reach the second vertex, then proceed from the second to the third vertex, and finally attempt to complete the path. Due to the absence of vision, blind individuals were likely impaired in considering the overall spatial information but rather walked the segments separately. As a result, creating a global spatial representation would be challenging for them (Setti et al., 2018). Conversely, in the guided condition, they followed the experimenter's guidance without any complex memory processing, likely making it much easier for them to remember the path. Similarly, in the linear trajectory, it is possible that participants focused on reaching the auditory stimulus without considering the entire trajectory they were walking. Our finding aligns with previous research where authors suggested that blind people do not perform well when moving alone because of the high spatial memory processing (Beni and Cornoldi, 1988; Cattaneo et al., 2008; Pearson et al., 2012). Moreover, in agreement with earlier research (Graso et al., 1999; Ruddle and Lessels, 2009; Campos et al., 2010), blind participants under the guidance of an experimenter efficiently employed idiothetic cues for spatial encoding, leading to lower error rates than in non-guided conditions.

To investigate whether the blind group's enhanced performance in the guided condition resulted from reduced error accumulation compared to the non-guided condition rather than differing information processing and, more in general, the impact of vision on this task, our study included blindfolded sighted participants. Our results revealed similar performance between the two conditions in this group. Furthermore, while the study lacks between-group testing, the non-guided condition shows remarkable consistency across groups compared to the guided condition. Specifically, the performance of blindfolded sighted individuals (around 100–110 cm for both conditions) aligns more closely, in absolute error terms, with the non-guided condition of blind individuals (78 cm and 95 cm) than with the guided condition (53 cm and 61 cm). Hence, it is probable that the enhanced performance of blind individuals in guided conditions stems from their adept processing of body-centered cues during guidance rather than from accumulating errors while walking, which could be attributed to their experience with rehabilitative training programs undertaken throughout their lives. Consequently, sighted individuals, unaccustomed to navigating without vision, interpret proprioceptive and body-centered cues less accurately compared to blind individuals. This underscores the importance of providing navigational aids specifically for the blind to support early contextual learning and enhance spatial representation over time.

Our second part, which is an administered questionnaire, gave an effective insight into the experience of blind individuals with the current technologies and their preferred feedback for future navigation systems. We observed no statistical significance that indicates that most of the blind people interviewed move alone when they go outside. Almost half of our participants use spatial navigation apps in conjunction with the cane, but they informally reported not being satisfied, primarily due to accuracy issues or concerns about draining their phone's battery. Moreover, in discussing the challenges blind individuals face, it was noted that many of them refrain from using numerous commercially available navigation aids. Issues were raised regarding the use of vibrating canes, with some participants citing discomfort due to heavy handles, while others expressed reservations about relying on navigator-based devices due to concerns about accuracy, power consumption, and safety. Many found vibrating canes to be overly sensitive to obstacles like sidewalks and lacking clear route guidance. Signal loss was also reported with GPS apps used for spatial navigation. Despite their benefits, some devices were criticized for occupying hands and requiring internet access. The abundance of information provided by sound-based applications overwhelmed several participants. Some cane-based devices presented complex feedback patterns, while others, like mobile apps, demonstrated inaccurate object detection. The cost was also a barrier for some aids. Additionally, our questionnaire revealed that most participants favored vibrational feedback over sound feedback for perceiving their surroundings. It's important to note that the apps and gadgets mentioned by blind individuals are based solely on their personal experiences, without scientific evidence to support their claims. In conclusion, our findings highlight the challenges the visually impaired face and underscore the need for more reliable and accessible navigation aids.

We acknowledge that our study has some limitations. First, the small sample size may limit the generalizability of our findings. Second, we did not include the vibrotactile or haptic condition for a better understanding of the influence of the sensory modality on the overall pattern of results. Third, we did not have any orientation and mobility experiences of our blind participants. Future research with larger sample sizes could validate and expand upon our results. Secondly, a comparison with the haptic or vibrotactile conditions would elucidate the effect of sensory modality.

## 5 Conclusion

Our study aimed to investigate two separate things: (1) the impact of guidance and non-guidance strategies in the encoding of the environment and (2) to understand the problems faced by blind individuals in current navigational strategies. Our experimental data indicated that encoding of the environment by blind people in non-guided conditions is not as accurate as it is in guided conditions. Our questionnaire results gave effective insights into avoiding the current navigational aids by blind people and their need for future navigational aids. These results will further help the studies to understand the mechanism of environment encoding and help the researchers to make a navigational aid by considering the problems faced by blind individuals and their needs.

## Data availability statement

The raw data supporting the conclusions of this article will be made available by the authors, without undue reservation.

## Ethics statement

The studies involving humans were approved by Comitato Etico, ASL 3, Genoa, Italy. The studies were conducted in accordance with the local legislation and institutional requirements. The participants provided their written informed consent to participate in this study.

## Author contributions

SS: Conceptualization, Data curation, Formal analysis, Investigation, Methodology, Visualization, Writing—original draft. WS: Conceptualization, Methodology, Writing—review & editing. CC: Conceptualization, Formal analysis, Writing—review & editing. SZ: Writing—review & editing. AD: Project administration, Supervision, Writing—review & editing. MG: Project administration, Supervision, Writing—review & editing.

## References

[B1] Amadeo M. B. Campus C. Pavani F. Gori M. (2019). Spatial cues influence time estimations in deaf individuals. IScience 19, 369–377. 10.1016/j.isci.2019.07.042 31415998 PMC6702436

[B2] Anson E. Ehrenburg M. Wei E. Bakar D. Simonsick E. Agrawal Y. (2019). Saccular function is associated with both angular and distance errors on the triangle completion test. Clini. Neurophysiol. 130, 2137–2143. 10.1016/j.clinph.2019.08.027 31569041 PMC6874399

[B3] Bai J. Liu D. Su G. Fu Z. (2017). “A cloud and vision-based navigation system used for blind people,” in Proceedings of the 2017 International Conference on Artificial Intelligence, Automation and Control Technologies [New York, NY: Association of computing Machinery (ACM)], 1–6.

[B4] Bates S. L. Wolbers T. (2014). How cognitive aging affects multisensory integration of navigational cues. Neurobiol. Aging 35, 2761–2769. 10.1016/j.neurobiolaging.2014.04.003 24952995

[B5] Beni R. d. Cornoldi C. (1988). Imagery limitations in totally congenitally blind subjects. J. Exp. Psychol. Learn. Mem. Cogn. 14:650. 10.1037/0278-7393.14.4.650 2972801

[B6] Blanco F. Travieso D. (2003). Haptic exploration and mental estimation of distances on a fictitious island: from mind's eye to mind's hand. J Visual Impair. Blin. 97, 298–300. 10.1177/0145482X0309700505

[B7] Brambring M. (1976). The structure of haptic space in the blind and sighted. Psychol. Res. 38, 283–302. 10.1007/BF00309777 1250939

[B8] Bredin J. Kerlirzin Y. Israël I. (2005). Path integration: is there a difference between athletes and non-athletes? Exp. Brain Res. 167:670–674. 10.1007/s00221-005-0251-3 16292571

[B9] Bregman A. S. Pinker S. (1978). Auditory streaming and the building of timbre. Can. J. Psychol./Revue canadienne de psychologie 32:19. 10.1037/h0081664 728845

[B10] Campos J. L. Byrne P. Sun H.-J. (2010). The brain weights body-based cues higher than vision when estimating walked distances. Eur. J. Neurosci. 31, 1889–1898. 10.1111/j.1460-9568.2010.07212.x 20584194

[B11] Cattaneo Z. Vecchi T. Cornoldi C. Mammarella I. Bonino D. Ricciardi E.. (2008). Imagery and spatial processes in blindness and visual impairment. Neurosci. Biobehav. Rev. 32, 1346–1360. 10.1016/j.neubiorev.2008.05.002 18571726

[B12] Chaccour K. Badr G. (2015). “Novel indoor navigation system for visually impaired and blind people,” in 2015 International Conference on Applied Research in Computer Science and Engineering (ICAR) (Beiriut: IEEE), 1–5.

[B13] Dennis I. Tapsfield P. (2013). Human Abilities: Their Nature and Measurement. New York: Psychology Press.

[B14] Dorado J. Figueroa P. Chardonnet J.-R. Merienne F. Hernández T. (2019). “Homing by triangle completion in consumer-oriented virtual reality environments,” in 2019 IEEE Conference on Virtual Reality and 3D User Interfaces (VR) (Osaka: IEEE), 1652–1657.

[B15] Ekstrom A. D. (2015). Why vision is important to how we navigate. Hippocampus 25, 731–735. 10.1002/hipo.22449 25800632 PMC4449293

[B16] Garcia S. Petrini K. Rubin G. S. Da Cruz L. Nardini M. (2015). Visual and non-visual navigation in blind patients with a retinal prosthesis. PLoS ONE 10:e0134369. 10.1371/journal.pone.0134369 26225762 PMC4520559

[B17] Gori M. Cappagli G. Baud-Bovy G. Finocchietti S. (2017). Shape perception and navigation in blind adults. Front. Psychol. 8:10. 10.3389/fpsyg.2017.00010 28144226 PMC5240028

[B18] Graso R. Glasauer S. george-franço. I. S Israël I. (1999). Replication of passive whole-body linear displacements from inertial cues: facts and mechanisms. Ann. N. Y. Acad. Sci. 871:345–366. 10.1111/j.1749-6632.1999.tb09197.x 10372084

[B19] Grieves R. M. Jeffery K. J. (2017). The representation of space in the brain. Behav. Processes 135, 113–131. 10.1016/j.beproc.2016.12.012 28034697

[B20] Harootonian S. K. Wilson R. C. Hejtmánek L. Ziskin E. M. Ekstrom A. D. (2020). Path integration in large-scale space and with novel geometries: comparing vector addition and encoding-error models. PLoS Comput. Biol. 16:e1007489. 10.1371/journal.pcbi.1007489 32379824 PMC7244182

[B21] Iachini T. Ruggiero G. (2010). The role of visual experience in mental scanning of actual pathways: Evidence from blind and sighted people. Perception 39, 953–969. 10.1068/p6457 20842972

[B22] Imrie R. Hall P. (2003). Inclusive Design: Designing and Developing Accessible Environments. Milton Park: Taylor & Francis.

[B23] Khusro S. Shah B. Khan I. Rahman S. (2022). Haptic feedback to assist blind people in indoor environment using vibration patterns. Sensors 22:361. 10.3390/s22010361 35009914 PMC8749676

[B24] Kosslyn S. M. Osherson D. N. (1995). An Invitation to Cognitive Science. Cambridge: MIT Press.

[B25] Koutakis P. Mukherjee M. Vallabhajosula S. Blanke D. J. Stergiou N. (2013). Path integration: Effect of curved path complexity and sensory system on blindfolded walking. Gait & Posture 37, 154–158. 10.1016/j.gaitpost.2012.06.027 22840893 PMC3485438

[B26] Koutsoklenis A. Papadopoulos K. (2011). Olfactory cues used for wayfinding in urban environments by individuals with visual impairments. J. Visual Impairm. Blindn. 105, 692–702. 10.1177/0145482X1110501015

[B27] Kuriakose B. Shrestha R. Sandnes F. E. (2022). Tools and technologies for blind and visually impaired navigation support: a review. IETE Techn. Rev. 39, 3–18. 10.1080/02564602.2020.1819893

[B28] Likova L. T. Cacciamani L. (2018). Transfer of learning in people who are blind: enhancement of spatial-cognitive abilities through drawing. J. Visual Impairm. Blindn. 112, 385–397. 10.1177/0145482X1811200405 33223582 PMC7677899

[B29] Loomis J. M. Klatzky R. L. Giudice N. A. (2018). “Sensory substitution of vision: Importance of perceptual and cognitive processing,” in Assistive Technology for Blindness and Low Vision (Boca Raton: CRC Press), 179–210.

[B30] Marder-Eppstein E. (2016). “Project tango,” in ACM SIGGRAPH 2016 Real-Time Live!, New York, NY: ACM.

[B31] Massiceti D. Hicks S. L. van Rheede J. J. (2018). Stereosonic vision: Exploring visual-to-auditory sensory substitution mappings in an immersive virtual reality navigation paradigm. PLoS ONE 13:e0199389. 10.1371/journal.pone.0199389 29975734 PMC6033394

[B32] Mittelstaedt H. (1999). The role of the otoliths in perception of the vertical and in path integration. Ann. N. Y. Acad. Sci. 871:334–344. 10.1111/j.1749-6632.1999.tb09196.x 10372083

[B33] Pearson D. De Beni R. Cornoldi C. (2012). “The generation, maintenance, and transformation of visuo-spatial mental images,” in Imagery, Language and Visuo-Spatial Thinking (New York: Psychology Press), 1–27.

[B34] Picinali L. Afonso A. Denis M. Katz B. F. (2014). Exploration of architectural spaces by blind people using auditory virtual reality for the construction of spatial knowledge. Int. J. Hum. Comput. Stud. 72, 393–407. 10.1016/j.ijhcs.2013.12.008

[B35] R Core Team (2013). R: A Language and Environment for Statistical Computing. Vienna: R Foundation for Statistical Computing.

[B36] Ran L. Helal S. Moore S. (2004). “Drishti: an integrated indoor/outdoor blind navigation system and service,” in Second IEEE Annual Conference on Pervasive Computing and Communications, 2004 (Orlando, FL: IEEE), 23–30.

[B37] Rieser J. J. Guth D. A. Hill E. W. (1986). Sensitivity to perspective structure while walking without vision. Perception 15, 173–188. 10.1068/p150173 3774488

[B38] Rogge A.-K. Hamacher D. Cappagli G. Kuhne L. Hötting K. Zech A.. (2021). Balance, gait, and navigation performance are related to physical exercise in blind and visually impaired children and adolescents. Experim. Brain Res. 239, 1111–1123. 10.1007/s00221-021-06038-3 33550429 PMC8068618

[B39] Ruddle R. A. Lessels S. (2009). The benefits of using a walking interface to navigate virtual environments. ACM Trans. Comp.-Human Interact. (TOCHI) 16, 1–18. 10.1145/1502800.1502805

[B40] Ruggiero G. Iachini T. (2010). The role of vision in the corsi block-tapping task: evidence from blind and sighted people. Neuropsychology 24:674. 10.1037/a0019594 20804256

[B41] Samany N. Delavar M. R. Saeedi S. Aghataher R. (2009). “3d continuous k-nn query for a landmark-based wayfinding location-based service,” in 3D Geo-Information Sciences (Cham: Springer), 271–282.

[B42] Santoro I. Murgia M. Sors F. Agostini T. (2020). The influence of the encoding modality on spatial navigation for sighted and late-blind people. Multisens. Res. 33, 505–520. 10.1163/22134808-20191431 31648190

[B43] Schwarze T. Lauer M. Schwaab M. Romanovas M. Böhm S. Jürgensohn T. (2016). A camera-based mobility aid for visually impaired people. KI-Künstliche Intelligenz 30:29–36. 10.1007/s13218-015-0407-732832221

[B44] Seemungal B. M. Glasauer S. Gresty M. A. Bronstein A. M. (2007). Vestibular perception and navigation in the congenitally blind. J. Neurophysiol. 97:4341–4356. 10.1152/jn.01321.2006 17392406

[B45] Setti W. Cuturi L. F. Cocchi E. Gori M. (2018). A novel paradigm to study spatial memory skills in blind individuals through the auditory modality. Sci. Rep. 8:13393. 10.1038/s41598-018-31588-y 30190584 PMC6127324

[B46] Shelton A. L. Yamamoto N. (2009). “Visual memory, spatial representation, and navigation,” in The Visual World in Memory (London: Psychology Press), 140–177.

[B47] Tinti C. Adenzato M. Tamietto M. Cornoldi C. (2006). Visual experience is not necessary for efficient survey spatial cognition: evidence from blindness. Quart. J. Experim. Psychol. 59, 1306–1328. 10.1080/17470210500214275 16769626

[B48] Wiener W. Lawson G. (1997). Audition for the traveler who is visually impaired. Foundat. Orientat. Mobilit. 2, 104–169.

[B49] Xie Y. Bigelow R. T. Frankenthaler S. F. Studenski S. A. Moffat S. D. Agrawal Y. (2017). Vestibular loss in older adults is associated with impaired spatial navigation: data from the triangle completion task. Front. Neurol. 8:173. 10.3389/fneur.2017.00173 28496432 PMC5406402

