# Corrigendum: How path integration abilities of blind people change in different exploration conditions

**DOI:** 10.3389/fnins.2024.1502268

**Published:** 2024-10-08

**Authors:** Shehzaib Shafique, Walter Setti, Claudio Campus, Silvia Zanchi, Alessio Del Bue, Monica Gori

**Affiliations:** ^1^Unit of Visually Impaired People (U-VIP), Italian Institute of Technology, Genova, Italy; ^2^Pattern Analysis and Computer Vision (PAVIS), Italian Institute of Technology, Genova, Italy

**Keywords:** blind navigation, path integration, shape completion, triangle completion task, spatial navigation, environment encoding, guided condition, feedback preference

In the published article, there was an error in the Funding statement. It was wrongly stated that no funding was received: “The author(s) declare that no financial support was received for the research, authorship, and/or publication of this article.” The correct Funding statement appears below.

